# Baseline plasma IL-18 may predict simvastatin treatment response in patients with ARDS: a secondary analysis of the HARP-2 randomised clinical trial

**DOI:** 10.1186/s13054-022-04025-w

**Published:** 2022-06-07

**Authors:** Andrew James Boyle, Peter Ferris, Ian Bradbury, John Conlon, Manu Shankar-Hari, Angela J. Rogers, Cecilia M. O’Kane, Daniel F. McAuley

**Affiliations:** 1 Wellcome-Wolfson Institute for Experimental Medicine, Centre for Experimental Medicine, Queen’s University Belfast, 97 Lisburn Road, Belfast, Northern Ireland; 2 Regional Intensive Care Unit, Royal Victoria Hospital, Grosvenor Road, Belfast, Northern Ireland; 3 Belfast, Northern Ireland; 4 Centre for Inflammation Research, The University of Edinburgh, The Queen’s Medical Research Institute, 47 Little France Crescent, Edinburgh, EH16 4TJ UK; 5 Division of Pulmonary, Allergy, and Critical Care Medicine, Department of Medicine, Stanford University, Stanford, CA USA

**Keywords:** Acute respiratory distress syndrome, Simvastatin, Inflammasome, Interleukin-18, Personalised medicine

## Abstract

**Background:**

Interleukin (IL)-18 is a marker of inflammasome activation, and high baseline plasma IL-18 is associated with increased mortality in patients with sepsis-induced ARDS. The aim of this analysis was to determine if simvastatin was associated with benefit in patients with ARDS and high plasma IL-18.

**Methods:**

In this secondary analysis of the HARP-2 study, we compared 28-day mortality and response to simvastatin according to baseline plasma IL-18 using cox proportional hazards analysis. Separately, monocyte-derived macrophages from healthy volunteers were pre-incubated with simvastatin or rosuvastatin before stimulation with ATP and LPS, and the effect on secreted IL-18 and IL-1β compared.

**Results:**

511 patients from HARP-2 had available data. High baseline plasma IL-18 (≥ 800 pg/ml) was associated with increased 28-day mortality (high IL-18 30.6% vs. low IL-18 17.5%; HR 1.89 [95% CI 1.30–2.73]; *p* = 0.001). Allocation to simvastatin in patients with high baseline plasma IL-18 was associated with a lower probability of 28-day mortality compared with placebo (24.0% vs 36.8%; *p* = 0.01). Finally, simvastatin, but not rosuvastatin, reduced stimulated macrophage secretion of IL-18 and IL-1β.

**Conclusion:**

In patients with high baseline plasma IL-18, simvastatin is associated with a higher probability of survival, and this effect may be due to reduced inflammasome activation. These data suggest that baseline plasma IL-18 may allow a personalised treatment approach by identifying patients with ARDS who could benefit from simvastatin therapy.

**Supplementary Information:**

The online version contains supplementary material available at 10.1186/s13054-022-04025-w.

## Introduction

The acute respiratory distress syndrome (ARDS) is a common clinical syndrome that is characterised by life-threatening acute hypoxaemic respiratory failure [1, 2]. ARDS results from excessive host inflammation that exacerbates the loss of alveolar-epithelial, capillary-endothelial barrier integrity [3].

The role of the inflammasome in the pathophysiology of ARDS is increasingly recognised. Activation of the NLR family pyrin domain containing 3 (NLRP3) inflammasome leads to caspase-1 activation which triggers cell death via pyroptosis and secretion of both IL-18 and IL-1β [4, 5]. In a murine model of hyperoxia-induced lung injury, mice deficient in NLRP3 had reduced lung injury compared with wild-type mice [6], whilst mice deficient in either caspase-1 or IL-18 had less alveolar neutrophilia, and lower lung injury scores, when exposed to injurious mechanical ventilation [7]. Critically ill patients with ARDS have higher plasma IL-18 than those without ARDS [7]. Plasma IL-18 also correlates with severity of hypoxaemia and mortality in patients with ARDS [8].

Simvastatin (in the HARP-2 trial) [9] and rosuvastatin (in the SAILS trial) [10] did not improve overall clinical outcomes in randomised controlled trials as therapies for ARDS. In a post hoc analysis of SAILS, baseline plasma IL-18 was positively associated with mortality and allocation to rosuvastatin was independently associated with increased odds of a rise in plasma IL-18 concentration [11], suggesting that rosuvastatin induced plasma IL-18. In a murine model of acute lung injury, it was demonstrated that pravastatin, a hydrophilic statin similar to rosuvastatin, increased pulmonary IL-1β and IL-18 *in vivo* and enhanced macrophage inflammasome activity *in vitro* [12]. This enhanced inflammasome activity was dependent on mitochondrial reactive oxygen species (mROS) generation in response to pravastatin. Although mROS generation has been reported to be differentially induced by hydrophilic and lipophilic statins [13], it is uncertain whether the pro-inflammasome activity observed is a statin class effect or specific to hydrophilic statins such as rosuvastatin and pravastatin.

In contrast to rosuvastatin, simvastatin is lipophilic and lipophilic statins have been shown to reduce NLRP3 inflammasome activation in endothelial cells [14]. Furthermore, latent class analysis of both the HARP-2 and SAILS trials demonstrated a difference in treatment effect; simvastatin improved survival in hyper-inflammatory ARDS, while rosuvastatin did not [15, 16]. This finding supports the concept that the effect of statins in ARDS may differ based on whether they are lipophilic or hydrophilic.

Therefore, we hypothesised that compared with rosuvastatin, simvastatin may have a different effect on outcomes in patients with ARDS who have higher baseline plasma IL-18 concentration. To address this, we performed a secondary analysis of the HARP-2 trial [9] and performed an *in vitro* study evaluating the effect of rosuvastatin and simvastatin in monocyte-derived macrophages.

## Materials and methods

This was a secondary analysis of the previously reported HARP-2 clinical trial [9]. In brief, 540 patients from 40 intensive care units across the UK and Ireland who fulfilled the American-European Consensus Conference definition for acute lung injury or ARDS [17] within the previous 48 h, were randomised to receive either simvastatin (80 mg once per day) or placebo for up to 28 days. Where available, baseline plasma samples were obtained from patients following trial enrolment, and this secondary analysis includes all patients from the HARP-2 trial with available baseline plasma samples. Ethical approval was granted by Queen’s University Belfast School Research Ethics Committee (ref. 14/06). During the consent process for HARP-2, written informed consent was obtained for storage and future analysis of samples. The primary outcome was 28-day mortality. Although ventilator-free days (VFDs) up to day 28 was the primary outcome in HARP-2 [9], we used 28-day mortality as the primary outcome for the purposes of this secondary analysis as it is a more patient-centred outcome to inform future trials.

Monocytes were isolated from the peripheral blood of healthy volunteers using density gradient centrifugation across a Ficoll-Paque gradient followed by adherence [18, 19], and were differentiated into macrophages (monocyte-derived macrophages (MDM)) by incubating in GM-CSF for 7 days [20]. Following a 24-h period of rest in fresh medium, macrophages were pre-treated for 4 h with 50 µM of either simvastatin or rosuvastatin calcium (Sigma-Aldrich, Germany). Cells were then treated with 100 ng/ml *Escherichia coli* 026:B6 lipopolysaccharide (LPS) for 3 h, before treatment with 2 mM adenosine triphosphate (ATP) (Sigma-Aldrich, Germany) for 30 min [21, 22]. An unstimulated control and a stimulated control (LPS + ATP) were also assessed. Cell culture supernatants were collected and processed for later analysis. MDMs from each patient were studied in each condition.

Total IL-18 and IL1β concentrations were measured in plasma or cell supernatants in duplicate by enzyme-linked immunosorbent assay (Duoset, R&D systems, Bio-Techne LTD, UK). If the coefficient of variation was > 10%, sample measurement was repeated in duplicate.

### Statistical methods

Descriptive statistics included calculation of proportions for categorical variables and mean (standard deviation (SD)) for continuous variables. The population of included patients was grouped by 28-day survivor status, and the statistical difference between these groups was calculated using chi-square test (or Fisher exact test) for discrete variables and by t test for continuous variables.

We used the previously reported threshold of IL-18 ≥ 800 pg/ml, demonstrated to be associated with increased mortality in patients with sepsis-associated ARDS, to define a high baseline plasma IL-18 [11]. The relationship between 28-day mortality and high baseline IL-18 was tested by proportional hazards regression using the actual time of death, censored at 28 days. Age, baseline APACHE II score, pre-randomisation partial pressure of oxygen to fraction of inspired oxygen (P/F) ratio, vasopressor use and having sepsis were pre-specified as clinically important variables to include in the models. These variables were selected to minimise the potential for confounding caused by increased plasma IL-18 with increasing age [23], the potential effect of baseline organ dysfunction and illness severity on plasma IL-18 concentration, and that there may be a difference in outcomes between patients with ARDS due to sepsis compared with ARDS from other causes [24]. SOFA score, baseline plasma C-reactive protein (CRP), plateau pressure and tidal volume were evaluated in an expanded univariate analysis to evaluate for an effect of baseline organ dysfunction, inflammation, pulmonary compliance and volutrauma. APACHE and SOFA were not both included in the multivariable models because the severity of illness variables from which they are calculated are broadly the same. The joint effect of simvastatin treatment and elevated IL-18 was studied by including the interaction term in the model and by subgroup analysis. As secondary analyses, area under the curve (AUC) for IL-18 and 28-day mortality was calculated, univariate and multivariable analysis was performed to assess the effect of this threshold on 28-day mortality, and correlation between baseline IL-18 and SOFA score at baseline, days 3 and 7 was calculated using Pearson’s r test.

Patients with incomplete data fields (e.g., missing APACHE II score) were excluded from the regression analyses. Results of logistic regression models are shown as odds ratios (OR) with 95% confidence intervals (CI) and p-value, and for proportional hazards regression as hazard ratios with 95% confidence intervals (CI) and p-value. Data were assumed missing completely at random, and this was confirmed using Little’s MCAR test (*p* = 0.85). These analyses were performed using the R statistical package (version 3.6.2).

*In vitro* data were normalised to the stimulated control and assessed using Kruskal–Wallis test with Dunn’s multiple comparison test to compare differences between the simvastatin and rosuvastatin groups, and between the statin groups and both the stimulated and unstimulated controls. The statistical analyses for the *in vitro* data were performed, and all the manuscript figures produced, using GraphPad Prism version 5.03.

## Results

Of the 540 patients recruited to the HARP-2 trial, 511 patients (95% of the enrolled trial population) were included in this analysis [9]. 28 patients had no baseline plasma sample available, and outcome data were unavailable for one patient. Of the included patients, 124 (24.3%) did not survive to day 28. Baseline characteristics are shown in Table 1. Non-survivors had a higher baseline plasma IL-18, a higher baseline APACHE II score, a lower pre-randomisation P/F ratio, were older, and more frequently receiving vasopressors.

**Table 1:** Baseline characteristics of included patients (stratified by survival status to day 28)

	Survivor (*N* = 387, 75.7%)	Non-survivor (*N* = 124, 24.3%)	*p*-value
Log baseline IL-18	6.71 (0.91)	7.06 (1.0)	< 0.001
Age (years)	51.51 (15.9)	59.40 (16.8)	< 0.001
Baseline APACHE II score^a^	17.95 (6.5)	21.87 (6.3)	< 0.001
Body mass index	27.4 (6.9)	26.7 (6.5)	0.31
Pre-randomisation *P*/*F* ratio (kPa)	17.41 (7.27)	16.14 (7.14)	0.05
Sepsis—*N* (%)	287 (74.2)	99 (79.8)	0.20
Vasopressor use—*N* (%)	237 (61.2)	95 (76.6)	0.002

Data presented as mean (standard deviation) unless otherwise stated

APACHE: Acute physiology and chronic health evaluation

^a^Data missing in 58 patients

Of the 511 patients included in this analysis, 453 had a complete dataset and were included in regression analyses. There was no difference in baseline characteristics between these patients and the 86 patients recruited to HARP-2 with available data that were not included (Additional file 1: Table E1, supplementary appendix). Similarly, there was no difference in 28-day mortality between patients included and excluded in regression analyses (24% vs. 27%; *p* = 0.69).

### High baseline plasma IL-18 is associated with increased 28-day mortality

**Fig. 1:**
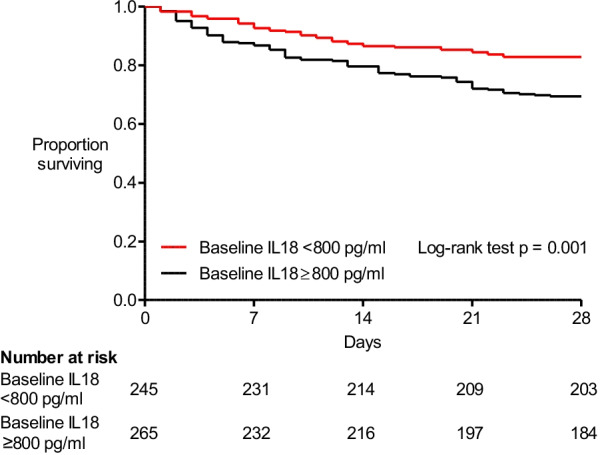
28-day survival dichotomised by baseline plasma IL-18. Kaplan–Meier curve of survival from enrolment to day 28. Survival was higher in patients with low plasma IL-18 at baseline (HR 1.89 [1.30–2.73]; log-rank test *p* = 0.001)

265 patients (51.9%) were categorised as having high baseline plasma IL-18, based on a previously defined threshold of ≥ 800 pg/ml [11], and 28-day mortality was higher in these patients than in patients with low baseline plasma IL-18 (high 30.6% vs. low 17.5%). The hazard ratio (HR) for death by day 28 associated with a high baseline plasma IL-18 was 1.89 (95% CI 1.30–2.73; cox proportional hazards *p* = 0.001) (Fig. 1). When adjusted for age, baseline APACHE II score, pre-randomisation P/F ratio, sepsis and vasopressor use, the hazard ratio for death by day 28 remained elevated for high baseline plasma IL-18 (1.61 [1.08–2.40]; *p* = 0.02) (Additional file 1: Table E2). Similarly, there were fewer ventilator-free days in patients with high baseline plasma IL-18 compared with those with low baseline plasma IL-18 (10.1 (10.1) vs. 14.1 (9.9); *p* < 0.001).

When analysing IL-18 as a (log-transformed) continuous variable, the association with 28-day mortality was maintained in univariate (HR 1.35 [1.15–1.58; *p* < 0.001) and multivariable analysis (1.30 [1.08–1.57]; *p* = 0.01), after adjustment for randomisation to simvastatin and baseline characteristics (Additional file 1: Table E3).

AUC analysis identified that plasma IL-18 concentration of 1014 pg/ml had the optimal specificity for 28-day mortality in this cohort (Additional file 1: Table E4). 216 (42.2%) of patients had a baseline plasma IL-18 ≥ 1014 pg/ml, and of these patients 70 (32%) did not survive to 28-day days. In both univariable and multivariable analysis, a plasma IL-18 threshold ≥ 1014 pg/ml remained associated with 28-day mortality (adjusted odds ratio (OR) 2.08 (1.30–3.33; *p* = 0.002) (Additional file 1: Table E5).

Finally, there was no relationship between baseline plasma CRP, plateau pressure or tidal volume with 28-day mortality, whilst SOFA score demonstrated a similar effect to that observed with APACHE-II score (Additional file 1: Table E6).

### Simvastatin is associated with reduced mortality in patients with high baseline plasma IL-18

**Fig. 2:**
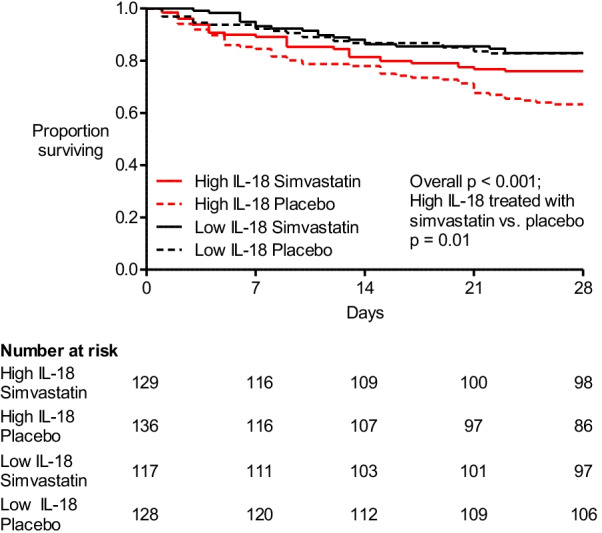
28-day survival stratified by baseline IL-18 and treatment simvastatin vs. placebo). Kaplan–Meier curve of survival from enrolment to day 28. Overall *p* value < 0.001. Patients with high baseline IL-18 (≥ 800 pg/ml) treated with simvastatin vs placebo *p* = 0.01; patients with low baseline IL-18 (< 800 pg/ml) treated with simvastatin vs. placebo *p* = 0.75. Interaction of simvastatin and high baseline IL-18 *p* = 0.19

Of the patients with high baseline IL-18, 129 (48.7%) were randomised to receive simvastatin in HARP-2. In patients with high baseline IL-18, survival to day 28 was higher in patients randomised to simvastatin compared with placebo (24.0% vs 36.8% 28-day mortality; *p* = 0.01). In contrast, 118 (48.0%) patients with low baseline IL-18 were randomised to receive simvastatin, and there was no difference in 28-day survival between these patients and those randomised to receive placebo (17.8% vs. 17.2% 28-day mortality; *p* = 0.75) (Fig. 2).

In multivariable logistic regression analysis, in patients with high baseline plasma IL-18, allocation to simvastatin was associated with reduced odds of 28-day mortality (adjusted OR 0.39 (0.20–0.73; *p* = 0.004) (Additional file 1: Table E7). However, formal statistical testing for an interaction between simvastatin and baseline plasma IL-18 was not significant (*p* = 0.19). The results were similar when a plasma IL-18 threshold of ≥ 1014 pg/ml was used (*p* = 0.49).

### Baseline plasma IL-18 is positively correlated with SOFA score during the first 7 days of ARDS

To understand whether baseline plasma IL-18 may be related to systemic organ dysfunction in the first 7 days of ARDS, correlation between baseline plasma IL-18 and SOFA score at baseline, days 3 and 7 was calculated. At each timepoint there was a positive correlation between baseline plasma IL-18 and SOFA score (baseline (Pearson *r* 0.28 [0.19, 0.6]; *p* < 0.0001); day 3 (0.26 [0.17, 0.34]; *p* < 0.0001); day 7 0.20 [0.09, 0.30]; *p* = 0.001) (Additional file 1: Figure S1).

### Simvastatin, but not rosuvastatin, reduces inflammasome activation in MDMs

**Fig. 3:**
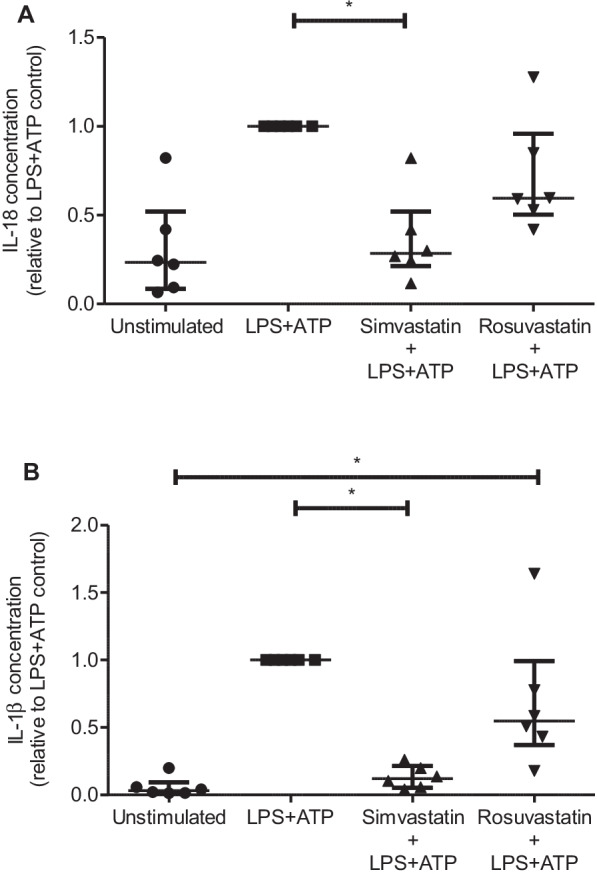
Effect of statin pre-treatment on monocyte-derived macrophage inflammasome activation. Monocyte-derived macrophages were pre-treated with either 50 µM simvastatin or 50 µM rosuvastatin for 4 h before stimulation with 100 ng/ml LPS and 2 mM ATP. In contrast to rosuvastatin, simvastatin significantly reduced the production of both (**A**) IL-18 and (**B**) IL-1β by MDMs (*p* < 0.05). When compared to the unstimulated control, rosuvastatin pre-treatment was associated with significantly higher IL-1β production (*p* < 0.05). Data analysed using Kruskal–Wallis test with Dunn’s multiple comparison test for between group differences. * = *p* < 0.05. *N* = 6 for all groups

To understand the potential mechanism for the disparate findings between rosuvastatin (reported in SAILS [11]) and simvastatin (in HARP-2 that are reported here), the effects of statin therapy on inflammasome activation in macrophages, as measured by IL-18 and IL-1β release, was assessed. Macrophages were pre-treated with either rosuvastatin or simvastatin prior to stimulation with LPS and ATP to induce inflammasome activation. Simvastatin, but not rosuvastatin, significantly reduced secretion of IL-1β and IL-18 in response to LPS and ATP. Furthermore, macrophage secretion of IL-1β was significantly higher in the rosuvastatin pre-treatment group compared to the unstimulated control, whilst there was no difference between the simvastatin pre-treatment group and the unstimulated control (Fig. 3).

## Discussion

In this secondary analysis of the HARP-2 clinical trial [9], we confirmed that baseline plasma IL-18 (≥ 800 pg/ml) is associated with mortality in a broad population of patients with ARDS, and present the novel finding that simvastatin may be associated with reduced mortality in patients with ARDS who have high baseline plasma IL-18 (≥ 800 pg/ml). Furthermore, we performed mechanistic studies that may explain the disparate effects of rosuvastatin and simvastatin, showing that in contrast to rosuvastatin, simvastatin pre-treatment reduced MDM inflammasome activity in human macrophages.

In addition to the findings from SAILS [11], enhanced inflammasome activity has been previously been identified in patients with ARDS when compared with patients with sepsis, systemic inflammatory response syndrome, and healthy controls [7]. Our data support the theory that early plasma IL-18 concentration is a prognostic marker in ARDS, which is also consistent with previous data demonstrating that elevated serum IL-18 in the early phase of ARDS is associated with increased mortality at 90 days [8]. Furthermore, we demonstrate that there is a positive correlation between baseline plasma IL-18 and SOFA score at baseline, days 3 and 7. Although the correlation was weak, these data support further investigation as a component of further study of IL-18 as a prognostic marker for ARDS.

Simvastatin has been studied as a therapy for ARDS because of its pleiotropic effects [25]. Whilst pre-clinical and early phase clinical trial data supported an anti-inflammatory effect in healthy subjects with experimental lung injury [19], and patients with ARDS [26], when studied in a large randomised clinical trial, there was no significant effect on clinical outcomes in patients with ARDS [9]. Heterogeneity in ARDS biology is increasingly recognized, with numerous ARDS cohorts best described by 2 phenotypes rather than one [27–29]. We have previously shown that the hyperinflammatory sub-phenotype (identified as part of latent class analysis) may benefit from simvastatin therapy [15], suggesting that a personalised approach to therapy is likely to be required in patients with ARDS. In this secondary analysis of the HARP-2 clinical trial, we present the novel finding that in patients with high baseline plasma IL-18, not measured as part of latent class analysis, randomisation to simvastatin was associated with reduced mortality at 28-days. Compared with latent class analysis, this single biomarker approach may be simpler for enrichment of future clinical trials for patients who may benefit from simvastatin. However, there was no significant interaction between simvastatin and high baseline plasma IL-18, and further studies are required to evaluate this relationship and the efficacy of using baseline plasma IL-18 as part of a personalised medicine approach to treatment.

Our *in vitro* data suggest there is a different effect on inflammasome activation between rosuvastatin and simvastatin, as we demonstrate that macrophages pre-treated with simvastatin, but not those pre-treated with rosuvastatin, had reduced inflammasome activation, as measured by IL-18 and IL-1β release, in response to LPS. Differences in the treatment effect between these statins have previously been demonstrated. For example, in the SAILS trial there was no treatment interaction between rosuvastatin and the hyper-inflammatory sub-phenotype [16], whilst there was a signal for benefit of simvastatin in patients with a hyper-inflammatory sub-phenotype in HARP-2 [15]. These data may reflect pharmacological differences between hydrophilic and lipophilic statins [12, 13]. Obesity can upregulate inflammasome activity [30], and although data on body mass index were not presented for patients recruited to SAILS [11] it is possible there may be differences in obesity, and therefore plasma IL-18 concentration, between the trial populations that may have influenced the effect of statin therapy. Finally, SAILS enrolled patients with sepsis-associated ARDS [10], whilst HARP-2 included patients irrespective of their risk factor for developing ARDS [9]. The attributable mortality from ARDS in patients with sepsis may be lower compared to patients with other aetiological risk factors [24]. This suggests the mechanisms implicated in determining mortality may differ according to the aetiological risk factor. Although the majority of patients in HARP-2 had sepsis, the difference in the recruited patient populations between the two studies may contribute to the difference in the observed effect between this study and the previously reported analysis of the SAILS study [11].

Our report has some limitations. First, it is not possible to infer causation from our data, and therefore we are unable to state with certainty that simvastatin reduces mortality of patients with ARDS who have high baseline plasma IL-18. Second, the robustness of our findings is limited by the absence of a significant interaction between simvastatin therapy and high baseline plasma IL-18, and this may relate to a lack of power to detect a statistically significant interaction. Whilst the magnitude of mortality difference of > 10% in patients with high IL-18 randomised to simvastatin would be clinically important, this lack of statistical significance of interaction makes these results more exploratory. Third, our measurement of total IL-18 may not fully reflect IL-18 activity because of the presence of IL-18 binding protein, which binds free active IL-18. In measuring total IL-18 it is plausible that we have measured both bound and free IL-18, and this measurement may not fully reflect IL-18 activity [31]. However, like sepsis, ARDS is characterised by systemic inflammation, and it is known that in sepsis although IL-18 binding protein is upregulated, free IL-18 remains elevated [32], suggesting that elevated total IL-18 is likely to still represent an increase in pro-inflammatory, free active IL-18. To better understand the relationship between total IL-18, IL-18 binding protein, inflammation, and clinical outcomes in patients with ARDS, further studies are required. Fourth, this is not a prospective study but a retrospective analysis of a clinical trial. Fifth, whilst the use of a pre-specified threshold to determine high plasma IL-18 may be considered a strength, we demonstrate that a baseline plasma IL-18 threshold of 1014 pg/ml had greater specificity for 28-day mortality in this population. Although the findings were similar, and the sensitivity for 28-day mortality was higher with the 800 pg/ml threshold, it remains uncertain what value of plasma IL-18 should be considered “high” in patients with ARDS and thus used in future studies. Sixth, the MDM data presented in this manuscript suggest that simvastatin attenuates macrophage-induced IL-18 and IL-1β release. These data represent a pre-treatment model which is different to both the SAILS and HARP-2 studies which evaluated statin therapy after ARDS onset, whilst prior statin use was an exclusion criteria [9, 10]. Furthermore, these *in vitro* findings were only evaluated in monocyte-derived macrophages. Although macrophages contribute to the pathophysiology of lung injury in ARDS [33], other cell types are also important in the development of ARDS [34]. It is likely that the effect of simvastatin in patients with high baseline plasma IL-18 is not exclusively mediated via macrophage activity and our findings should be considered hypothesis-generating. There remains a need for further studies in other cell types, and in addition to IL-18 secretion, measurement of IL-18 expression with RT-PCR would be valuable. In addition, the use of clinically relevant human models that may more accurately reflect ARDS biology [35], will further improve understanding of the role of IL-18 in ARDS.

## Conclusion

In summary, high baseline plasma IL-18 is independently associated with increased mortality in patients with ARDS, and patients with high plasma IL-18 at baseline had reduced 28-day mortality when randomised to simvastatin. These results require prospective validation in clinical trials to determine the potential for a personalised approach to ARDS that uses baseline plasma IL-18 to identify those who might benefit from simvastatin therapy.

## Supplementary Information

**Additional file 1.** Supplementary tables.

## Data Availability

The dataset used to generate this manuscript may be made available from the corresponding author on reasonable request.
