# Supplementary material for: Baseline plasma IL-18 may predict simvastatin treatment response in patients with ARDS: a secondary analysis of the HARP-2 randomised clinical trial
Source: Crit Care. 2022 Jun 7;26:164. doi: 10.1186/s13054-022-04025-w (PMC9175337; doi:10.1186/s13054-022-04025-w)
Supplement: Supplementary file 1 — Additional file 1. Supplementary tables. [file 13054_2022_4025_MOESM1_ESM.docx]

**Simvastatin is associated with reduced mortality in patients with ARDS and high baseline plasma IL-18**

Andrew J Boyle, Peter Ferris, Ian Bradbury, John Conlon, Manu Shankar-Hari, Angela J Rogers, Cecilia M O’Kane, Daniel F McAuley

**Online data supplement**

**A**

**B**

**C**


**Figure S1: Correlation between log baseline plasma IL-18 and SOFA score at baseline, day 3 and day 7.** There was a positive correlation between log baseline plasma IL-18 and SOFA score at baseline, day 3 and day 7 (baseline Pearson r 0.28 [0.19, 0.6]; p < 0.0001); day 3 (0.26 [0.17, 0.34]; p < 0.0001); day 7 0.20 [0.09, 0.30]; p = 0.001). Data analysed using Pearson’s r test and presented as r value (95% CI).

**Table E1**: Baseline characteristics of patients included and excluded from regression analyses

|  | **Included patients**  (N = 453) | **Excluded patients**  (N = 86) | **p-value** |
| --- | --- | --- | --- |
| **Age** (years) | 53.5 (16.6) | 52.7 (15.2) | 0.66 |
| **Baseline APACHE II score** ^a^ | 18.9 (6.7) | 17.3 (5.2) | 0.29 |
| **Body mass index** | 27.2 (6.7) | 26.6 (7.3) | 0.44 |
| **Pre-randomisation P/F ratio** (kPa) | 17.1 (7.4) | 17.0 (7.5) | 0.90 |
| **Sepsis ­**– N (%) | 338 (74.6) | 69 (80.2) | 0.27 |
| **Vasopressor use** – N (%) | 297 (65.6) | 59 (68.6) | 0.59 |

Data presented as mean (standard deviation) unless otherwise stated.

APACHE: Acute physiology and chronic health evaluation.

a: Data missing in 67 excluded patients

**Table E2**: Cox-proportional hazards model for 28-day mortality

|  | **Unadjusted analysis**  Hazard ratio  (95% CI; p-value) | **Multivariable analysis**  Hazard ratio  (95% CI; p-value) |
| --- | --- | --- |
| **High baseline plasma IL-18**  (≥800 pg/ml)  (N = 265) | 1.89  (1.30 – 2.73; p = 0.001) | 1.61  (1.08 – 2.40; p = 0.02) |
| **Age**  (53.4 [16.4] years) | 1.03  (1.02 – 1.04; p <0.001) | 1.02  (1.01 – 1.03; p = 0.003) |
| **Baseline APACHE II score**  (18.9 [6.7]) | 1.08  (1.05 – 1.11; p <0.001) | 1.05  (1.02 – 1.09; p <0.001) |
| **Pre-randomisation P/F ratio**  (17.1 [7.3] kPa) | 0.98  (0.95 – 1.00; p = 0.07) | 0.98  (0.95 – 1.00; p = 0.10) |
| **Sepsis**  (N = 386) | 1.36  (0.88 – 2.11; p = 0.17) | 0.94  (0.59 – 1.51; p = 0.81) |
| **Vasopressor use**  (N = 332) | 1.94  (1.28 – 2.94; p = 0.002) | 1.71  (1.06 – 2.74; p = 0.03) |

Number of patients in model = 453 (missing = 58)

APACHE: Acute physiology and chronic health evaluation.

Variables evaluated based on mean [standard deviation] unless otherwise stated

**Table E3**: Univariate and multivariable analysis for 28-day mortality

|  | **Univariate analysis**  Hazard ratio  (95% CI; p-value) | **Multivariable analysis**  Hazard ratio  (95% CI; p-value) |
| --- | --- | --- |
| **Log Baseline IL-18**  (per Log_2_ increase) | 1.35  (1.15 – 1.58; p <0.001) | 1.30  (1.08 – 1.57; p = 0.01) |
| **Simvastatin** | 0.75  (0.52 – 1.07); p = 0.10) | 0.58  (0.39 – 0.86; p = 0.01) |
| **Age**  (per unit increase in year) | 1.03  (1.02 – 1.04; p <0.001) | 1.02  (1.01 – 1.03; p = 0.004) |
| **Baseline APACHE II score** (per unit increase) | 1.08  (1.05 – 1.11; p <0.001) | 1.06  (1.03 – 1.09; p <0.001) |
| **Pre-randomisation P/F ratio** (per unit increase in kPa) | 0.98  (0.95 – 1.00; p = 0.07) | 0.97  (0.95 – 1.00; p = 0.06) |
| **Sepsis** | 1.36  (0.88 – 2.1; p = 0.17) | 0.95  (0.59 – 1.51; p = 0.83) |
| **Vasopressor use** | 1.94  (1.28 – 2.94; p = 0.002) | 1.64  (1.02 – 2.64; p = 0.04) |

Number of patients in model = 453 (missing = 58)

APACHE: Acute physiology and chronic health evaluation.

**Table E4:** Sensitivity and specificity for baseline plasma IL-18 concentration and 28-day mortality

|  | **Sensitivity** | **Specificity** |
| --- | --- | --- |
| Baseline plasma IL-18 ≥800 pg/ml | 61% | 52% |
| Baseline plasma IL-18 ≥1014 pg/ml | 46% | 61% |

**Table E5**: Univariate and multivariable analysis for 28-day mortality using baseline plasma IL-18 threshold of ≥1014 pg/ml

|  | **Univariate analysis**  Odds ratio  (95% CI; p-value) | **Multivariable analysis**  Odds ratio  (95% CI; p-value) |
| --- | --- | --- |
| **High baseline plasma IL-18**  (≥1014 pg/ml) | 2.17  (1.40 – 3.37; p < 0.001) | 2.08  (1.30 – 3.33; p = 0.002) |
| **Age**  (per unit increase in year) | 1.03  (1.02 – 1.05; p < 0.001 | 1.03  (1.01 – 1.04; p = 0.001) |
| **Baseline APACHE II score**  (per unit increase) | 1.10  (1.06 – 1.13; p < 0.001) | 1.07  (1.03 –1.11; p <0.001) |
| **Pre-randomisation P/F ratio**  (per unit increase in kPa) | 0.98  (0.95 – 1.01; p = 0.13) | 0.98  (0.95 – 1.01; p = 0.24) |
| **Sepsis** | 1.27  (0.76 – 2.13; p = 0.36) | 0.84  (0.51 – 1.55; p = 0.68) |
| **Vasopressor use** | 2.36  (1.42 – 3.92; p <0.001) | 1.72  (1.04 – 3.10; p = 0.04) |

Number of patients in model = 453 (missing = 70)

APACHE: Acute physiology and chronic health evaluation.

Variables evaluated based on mean [standard deviation] unless otherwise stated

**Table E6:** Univariate analysis for additional covariates and 28-day mortality

|  | **Univariate analysis**  Odds ratio  (95% CI; p-value) |
| --- | --- |
| **Baseline CRP**  (per unit increase) | 1.00  (1.00 – 1.00; p = 0.18) |
| **Baseline plateau pressure**  (per unit increase) | 1.02  (0.98 – 1.07; p = 0.31) |
| **Baseline SOFA score**  (per unit increase) | 1.17  (1.09 – 1.26; p < 0.001) |
| **Baseline tidal volume**  (per unit increase in ml / kg (ideal body weight)) | 1.00  (0.95 – 1.06; p = 0.93) |

**Table E7**: Univariate and multivariable analysis evaluating 28-day mortality in patients with baseline plasma IL-18 ≥800 pg/ml

|  | **Univariate analysis**  Odds ratio  (95% CI; p-value) | **Multivariable analysis**  Odds ratio  (95% CI; p-value) |
| --- | --- | --- |
| **Simvastatin** | 0.54  (0.32 – 0.92; p = 0.03) | 0.39  (0.20 – 0.73; p = 0.004) |
| **Age** (per unit increase in year) | 1.03  (1.02 – 1.05; p <0.001) | 1.02  (1.00 – 1.05; p = 0.03) |
| **Baseline APACHE II score** (per unit increase) | 1.10  (1.04 – 1.15; p <0.001) | 1.09  (1.03 – 1.16; p = 0.002) |
| **Pre-randomisation P/F ratio** (per unit increase in kPa) | 0.99  (0.96 – 1.03; p = 0.74) | 0.99  (0.95 – 1.03; p = 0.73) |
| **Sepsis** | 1.02  (0.54 – 2.01; p = 0.95) | 0.56  (0.26 – 1.22; p = 0.14) |
| **Vasopressor use** | 1.74  (0.96 – 3.25; p = 0.08) | 1.89  (0.90 – 4.16; p = 0.10) |

Number of patients in model = 232 (missing = 33)

APACHE: Acute physiology and chronic health evaluation.
